# An Innovative Approach for Breast Conservation in a Patient With Multicentric Upper Inner and Central Quadrant Breast Tumors

**DOI:** 10.7759/cureus.94948

**Published:** 2025-10-19

**Authors:** Satyajit Kundu, Srijita Saha, Divya Dahiya, Ishita Laroiya

**Affiliations:** 1 Endocrine and Breast Surgery Unit, Department of General Surgery, Postgraduate Institute of Medical Education and Research, Chandigarh, IND; 2 Department of General Surgery, Postgraduate Institute of Medical Education and Research, Chandigarh, IND

**Keywords:** breast cancer, breast conservation surgery, chest wall perforator flaps, micap flap, multicentric tumour, oncoplastic breast surgery

## Abstract

Oncoplastic breast surgery expands the number of options for conservative breast cancer treatment. Traditionally, patients with multicentric tumors, especially those in the upper and central quadrant, were offered mastectomy. Recent evidence supports the safety and feasibility of breast conservation surgery even in patients with multifocal and multicentric breast cancer. However, there remains a challenge to achieve wide local excision of tumors while maintaining a good cosmetic outcome, especially in central and upper inner quadrant tumors.

Here, we present a case where a patient with multicentric upper inner and central quadrant tumors was managed with breast-conserving surgery: wire bracketing of the lesions with wide local excision with nipple-areolar complex, followed by volume replacement and areolar reconstruction with medial intercostal artery perforator (MICAP) flap. The patient had a satisfactory cosmetic outcome with no complications. She continues to be disease-free and satisfied with the postoperative result at 15 months post operation.

## Introduction

Some aspects of breast surgery remain challenging, limiting our capacity to provide the best care to our patients. Upper inner quadrant (UIQ) continues to be a challenging area for oncoplastic breast surgeons, particularly in patients with small/moderate breasts. This region, often referred to as a surgical "no-man’s land," offers limited adjacent tissue mobility and relatively inelastic skin, restricting the feasibility of local flap rearrangements. Consequently, wide excisions in this area commonly result in contour deformities and distortion of the nipple-areolar complex (NAC), thereby compromising cosmetic outcomes. Traditionally, extreme caution has been exercised when considering breast-conserving surgery (BCS) for UIQ tumors [1].

Current research suggests that breast conservation can be safely done for multifocal and multicentric breast cancer (where it is technically possible and satisfactory cosmetic outcomes can be achieved) [2]. Hamdi et al. first described intercostal artery perforator (ICAP) flaps for partial breast reconstruction [3]. Chest wall perforator flaps (CWPFs), which are muscle-sparing flaps using small blood vessels near the chest wall, outperform standard options such as latissimus dorsi (LD) flaps because donor site morbidity is minimized [4]. Here, we present a case of a female who underwent BCS with medial intercostal artery perforator (MICAP) flap reconstruction for multicentric (MC) upper inner and central quadrant breast tumors. Thus, a MICAP flap can enable breast conservation in a scenario traditionally managed by mastectomy, offering oncological safety with cosmetic satisfaction.

## Case presentation

A 48-year-old peri-menopausal female with no known comorbidities presented to our institute with a painless, gradually progressive lump in the UIQ of the left breast and associated with spontaneous intermittent bloody nipple discharge in the left breast for two months. There was no history of prior breast disease and no significant family history. The patient’s BMI was 32.6, and she had a D cup bra size. On clinical examination, an ill-defined firm lump of size 2 x 2 cm was palpable in the UIQ of the left breast, 1 cm away from the NAC, with the presence of skin tethering over the lump. The lump was not fixed to the chest wall/pectoralis major. No axillary lymphadenopathy was present.

Investigations

**Figure 1:**
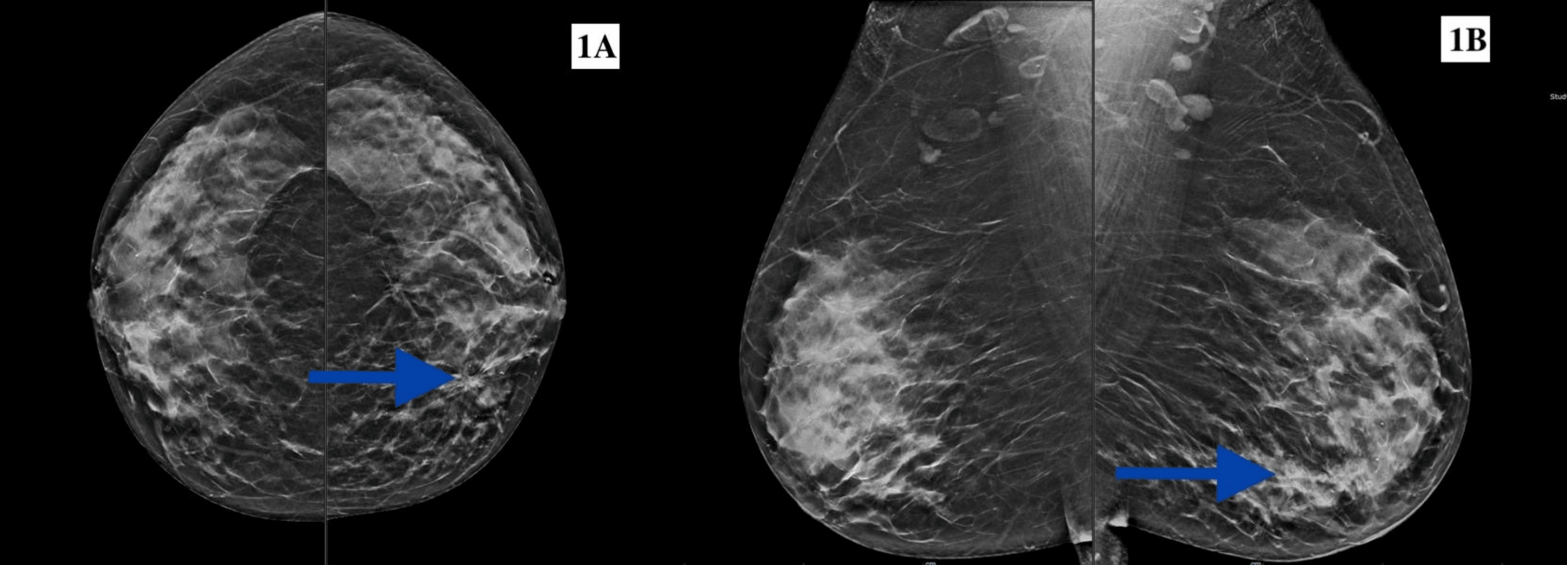
Mammography showing mediolateral (A) and craniocaudal view (B). The blue arrow shows an ill-defined mass with indistinct margins in the central quadrant of the left breast with associated architectural distortion. Multicentricity was better appreciated on ultrasound breast images.

Mammography showed American College of Radiology (ACR) grade C density (Figure 1). There was an ill-defined mass with indistinct margins in the central quadrant of the left breast. Ultrasonography of the left breast revealed an ill-defined hypoechoic lesion measuring 15 x 8 mm in the nine o'clock position, involving the central quadrant with angular margins. Also, two satellite lesions were seen at the nine o'clock and 11 o'clock positions in the UIQ, 4 cm from the NAC. A few benign-appearing lymph nodes were seen in the left axilla. The final impression was a multicentric tumor of the left breast: Breast Imaging Reporting and Data System (BIRADS) 4. The right breast was normal. Clinical staging was cT1(m)N0M0.

Core needle biopsy was taken from the lump in the nine o'clock position, which showed invasive breast carcinoma of no special type, grade I, estrogen receptor (ER) 2+ positivity in 60% of tumor cells (Allred score = 6/8), progesterone receptor (PR) negative, HER2/neu negative, with Ki-67 of 5%.

Treatment

She was given the option of lumpectomy and MICAP flap reconstruction versus an oncoplastic reduction mammoplasty along with a symmetrization procedure on the opposite side. She chose the former option as she did not want to undergo a surgical procedure on the opposite breast. She underwent wire-guided bracketing and wide local excision with NAC, along with sentinel lymph node biopsy (SLNB) with MICAP flap reconstruction.

**Figure 2:**
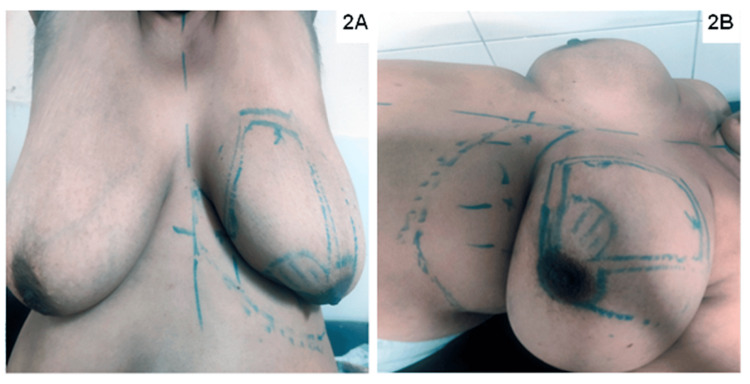
Marking of tumor and satellite lesions, along with the medial intercostal artery perforator flap in the sitting (A) and supine position (B). Breast contour changes with position, and flap width is assessed by the pinch test, which is best done in the sitting position.

Preoperative marking involved the following steps: initially, the inframammary fold (IMF), breast meridian, and midline were marked. Next, the excision area was carefully defined. The medial intercostal artery perforator, which is typically 1.5 to 2.5 cm from the midline and 1 to 2 cm below the IMF, was identified and marked using Doppler prior to surgery (Figure 2).

**Figure 3:**
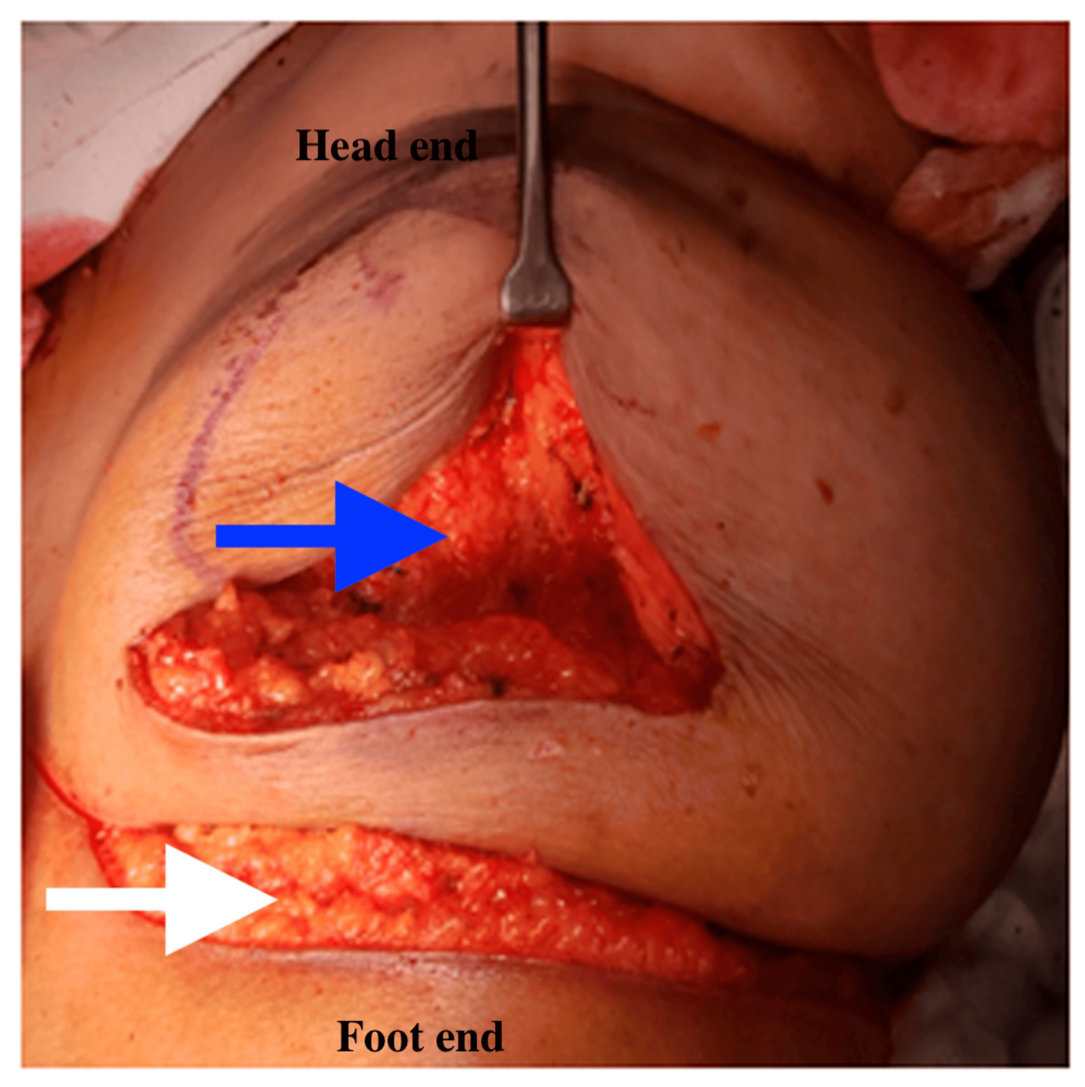
Tumor bed after excision (blue arrow) and white arrow showing the infra-mammary fold.

The operating procedure was as follows: preoperative wire-guided localization and bracketing of two satellite lesions were carried out. A SLNB was performed utilizing the dual technique, resulting in the identification of three sentinel lymph nodes (SLNs), which were sent for frozen section analysis and found to be negative. A periareolar incision with medial extension was carried out to encompass the skin tethering area. A wide local excision with the NAC was performed, removing roughly 30% of the breast volume. Lumpectomy was performed with appropriate margins (Figure 3). The margins were confirmed using specimen mammography, which revealed that the tumor and both the satellite lesions had clear margins.

**Figure 4:**
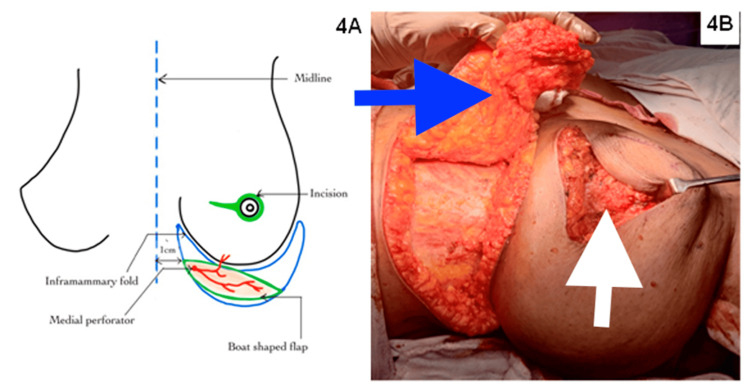
Designing (A) and harvesting (B) of the medial intercostal artery perforator (MICAP) flap. The blue arrow shows the MICAP flap, and the white arrow shows the tumor bed after excision. The MICAP flap is harvested based on the medial intercostal artery perforators. The superior border is the infra-mammary fold, and the inferior border is decided by the pinch test, which is best done in the supine position, medially till the parasternal area, and laterally till the anterior axillary line.

**Figure 5:**
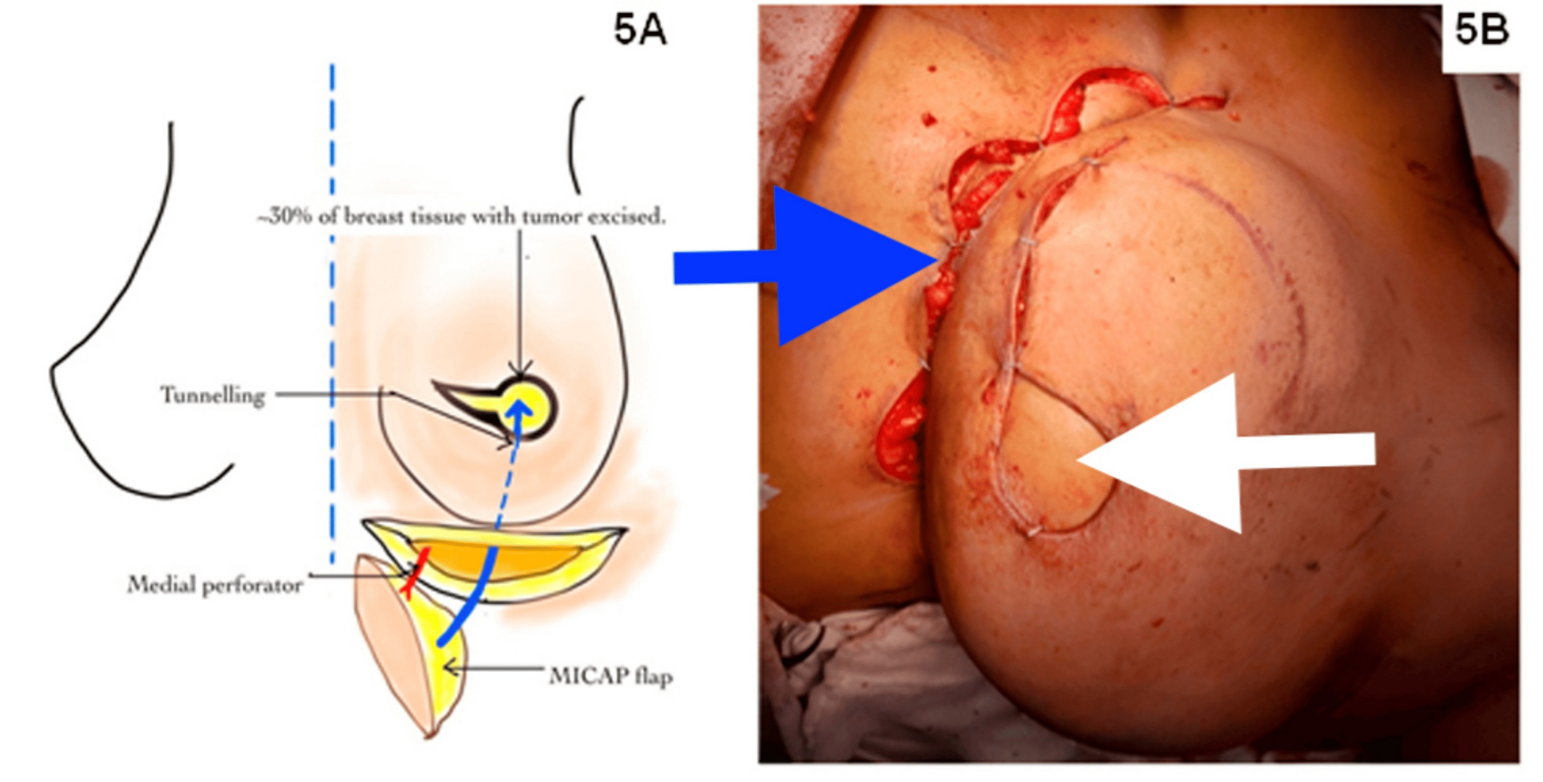
Medial intercostal artery perforator (MICAP) flap tunneling (A) and inset (B). The blue arrow shows the infra-mammary fold, and the white arrow shows the skin paddle of the MICAP flap.

A boat-shaped flap was defined, with the IMF as the upper boundary and the medial border around 1 cm from the midline (Figure 4A). Then, the MICAP flap was harvested based on medial internal anterior intercostal artery perforators (MICAP), as shown in Figure 4B. The flap was raised laterally from the anterior axillary line and medially to the medial third, utilizing the internal anterior intercostal artery perforators (AICAP). The flap was de-epithelialized, tunneled, and inset into the defect (Figure 5). The total operative time was four hours, and blood loss was 50 ml.

Outcome and follow-up

**Figure 6:**
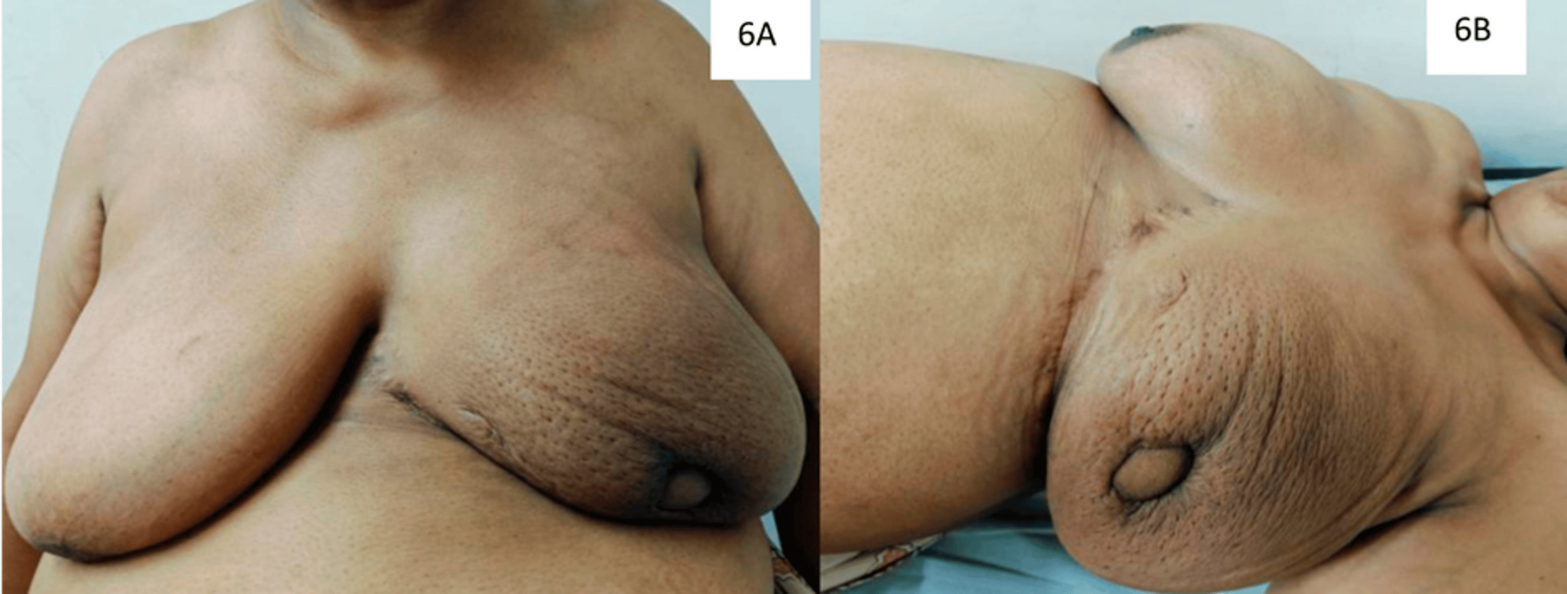
Eight months postoperative image showing reconstructed breast and radiation-induced skin changes in the sitting (A) and supine positions (B). The overall cosmetic outcome is good with a slight difference in the size and shape of the breast.

The patient did not have any postoperative complications and was discharged on the second postoperative day. Final pathological examination revealed infiltrating ductal carcinoma grade 1 and multiple foci of invasive carcinoma (number of foci: three with a maximum size of 1.8 x 1.5 x 1.2 cm). All margins were negative, and 0/6 lymph nodes were negative. As per the multidisciplinary team decision, the patient was given adjuvant radiotherapy and tamoxifen (planned for 10 years). The patient is currently 15 months post operation and continues to be disease-free and without any complaints. She is very satisfied with the result of her surgery (Figure 6). The patient-reported outcome measures (PROMs) assessment was done with a questionnaire adapted from the UK National Mastectomy and Breast Reconstruction Audit (NMBRA) study (Table 1) [5].

**Table 1:** Patient-reported outcome measures. Questionnaire adapted from the National Mastectomy and Breast Reconstruction Audit (NMBRA) study [5].

Information domain	At 3 months	At 6 months	At 12 months
Patient satisfaction with the result of the surgery	Very satisfied	Very satisfied	Very satisfied
Emotional well-being (emotionally confident)	All of the time	All of the time	All of the time
Pulling sensation in the abdomen	Nil	Nil	Nil
Pain at surgery and the donor site	Sometimes	Sometimes	Occasional

## Discussion

Breast conservation therapy (BCT) is now a viable alternative for multicentric/multifocal (MC/MF) carcinomas of the breast, and the presence of MC/MF alone does not necessitate mastectomy. In the St. Gallen/Vienna consensus 2023, the panel for the first time recommended breast conservation for multicentric disease [6]. Oncoplastic surgery combines oncologic and reconstructive surgical approaches to improve the cosmesis of breast cancer patients.

**Figure 7:**
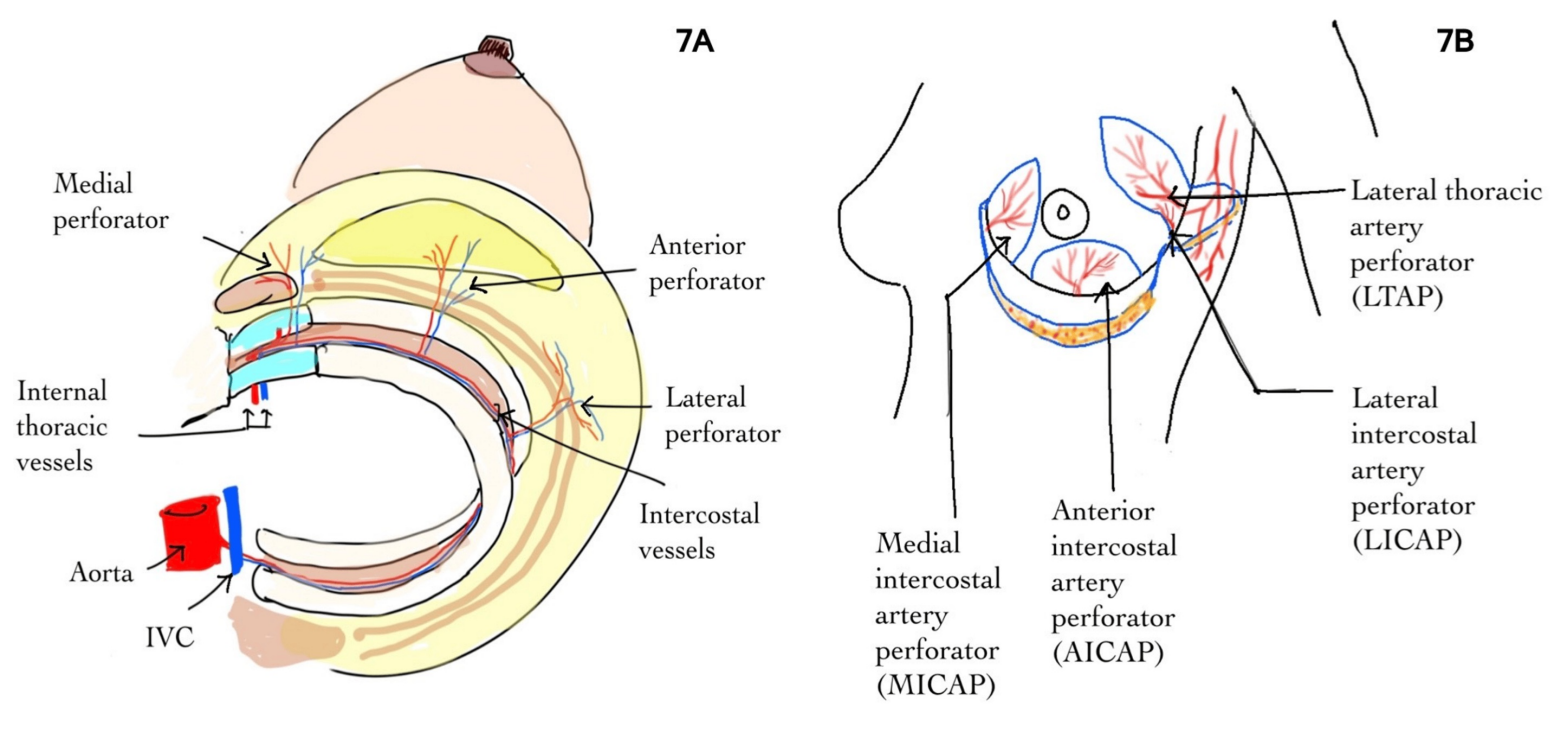
Different intercostal artery perforators (A) and various types of chest wall perforator flaps (B). MICAP flap for medial quadrant tumors, AICAP flap for six o'clock tumors, and LICAP flap for outer quadrant tumors. IVC: inferior vena cava.

CWPFs are muscle-sparing fasciocutaneous flaps that are gaining popularity. This progress has been facilitated by a better understanding of the regional distribution of ICAPs in the chest wall [7,8]. Different intercostal artery perforators and various types of chest wall perforator flaps are shown in Figure 7. The advantages include avoiding a mastectomy and providing skin coverage for reconstruction. In comparison to the LD flap, CWPF surgery has fewer complications, such as seroma, wound dehiscence, and loss of shoulder function [9].

While some UIQ tumors in moderate/large breasts can be treated with oncoplastic methods such as batwing mastopexy and matrix rotation mammoplasty [1,10], the majority of these produce suboptimal cosmetic results and are insufficient when the tumor/breast size ratio is unfavorable. Also, these need opposite breast symmetrization, which may be unacceptable to some women. A review of the literature shows that while CWPFs have been reported for breast reconstruction [11-14], there are no published cases describing their use in multicentric tumors involving the upper inner and central quadrants. Hence, in this case, a novel BCS approach combined with a MICAP flap for multicentric upper inner and central quadrant breast cancer was used, which resulted in an acceptable PROM while maintaining oncological safety.

The MICAP flap offers clear benefits, including less donor site morbidity, good cosmetic outcomes, and versatility. But it does have challenges. Its success depends on thorough preoperative planning and precise intraoperative technique, since the flap relies on small, variable perforators from the medial intercostal vessels. This makes dissection more demanding than with other perforator flaps and adds a learning curve.

The flap’s short arc of rotation also limits its use mainly to the UIQ and sometimes the central breast. To improve reliability, surgeons should use preoperative Doppler to locate good perforators, design the flap carefully within the safe vascular territory, handle pedicles gently with minimal cautery, and preserve nearby vessels. Checking the flap’s reach before division helps avoid pedicle tension. With these precautions, MICAP flaps can be a safe and effective option for UIQ breast reconstruction.

## Conclusions

MC/MF tumors are not a contraindication of BCS. CWPFs have become the technique of choice for partial breast reconstruction in carefully chosen patients. The case demonstrated clear margins, complication-free recovery, and high patient satisfaction, supporting the conclusion that the MICAP flap can provide oncological safety and acceptable cosmesis in multicentric upper inner and central breast tumors. However, since this is a single case, the generalizability is limited and should be more clearly emphasized.
